# Efficacy of L-arginine and Pycnogenol ® in the treatment of male erectile dysfunction: a systematic review and meta-analysis

**DOI:** 10.3389/fendo.2023.1211720

**Published:** 2023-10-04

**Authors:** Yuting Tian, Qian Zhou, Wen Li, Meixi Liu, Qing Li, Qiu Chen

**Affiliations:** ^1^ Hospital of Chengdu University of Traditional Chinese Medicine, Chengdu, China; ^2^ School of Clinical Medicine, Chengdu University of Traditional Chinese Medicine, Chengdu, China

**Keywords:** erectile dysfunction, Pycnogenol ®, L-arginine, efficacy, meta-analysis

## Abstract

**Background:**

The objective of this meta-analysis was to review clinical trials of the combination of Pycnogenol ® and L-arginine (PAL) in the treatment of erectile dysfunction in men and to observe the effect of PAL combined therapy on sexual function in patients with erectile dysfunction (ED), and we hope to provide more choices of drugs for treating patients with ED.

**Methods and analysis:**

The study was constructed according to the Preferred Reporting Items for Systematic Reviews and Meta-Analysis guidelines. We searched seven databases from inception to 15 February 2023, for a comprehensive search of clinical trials using relevant keywords. Continuous variables in this meta-analysis were calculated using the mean difference and 95% confidence interval. All relevant statistical analyses were performed using RevMan v. 5.4 software.

**Results:**

Three studies with 184 patients were included in the present meta-analysis. There were no significant differences in the basic characteristics of the included studies. The results of the current meta-analysis showed that there were significant differences in the international index of erectile function scores (erectile domain), intercourse satisfaction scores, orgasmic function scores, overall satisfaction scores, and sexual desire scores between the combination treatment group and the control group. There was no significant difference in improving the testosterone levels between the two groups.

**Conclusion:**

These results indicate that the combination of PAL may have a significant effect on improving sexual function in patients with mild to moderate ED. This study will provide clinicians with more options for treating patients with ED. More randomized controlled trials are needed in the future to further demonstrate the effect of combination therapy on sexual function in patients with ED.

**Systematic review registration:**

https://www.crd.york.ac.uk/PROSPERO/#myprosperoUnique, Identifier: CRD42023411781.

## Introduction

Erectile dysfunction (ED) refers to the persistent or recurring phallic erection that is difficult to obtain or maintain enough to meet sexual needs (1, 2). ED is a very common and increasingly serious male disease worldwide, and the risk of the disease is increasing with age (3, 4). It is estimated that nearly 140 million men worldwide are suffering from varied degrees of ED, and ED is expected to affect 322 million people by 2025 (5, 6). There are many reasons for ED. Age, smoking, psychological disorders, unfavorable socio-economic conditions, depression and psychiatric illness, cardiovascular diseases, diabetes, hypercholesterolemia (7), obstructive sleep apnea (8), and celiac disease (9) are all risk factors for ED. ED is often caused by other diseases, so it is easy to be ignored.

Priapism is a normal physiological response (10). When the body experiences sexual stimulation, paraspathetic nerve activity from the sacral part of the spinal cord triggers a series of reactions, releasing nitric oxide (NO) and increasing the intracellular cyclic guanosine monophosphate (cGMP). NO is essential for achieving an erection. NO is the messenger cell of penile erection, which is produced by neuronal NO synthase (NOS) of penile non-adrenergic, non-cholinergic nerve terminals and endothelial NOS (E-NOS) of penile artery endothelial cells. After NO production, it binds to guanylate cyclase in the smooth muscle cells of the corpus cavernosum to generate cGMP. Increased cGMP leads to relaxation of vascular smooth muscle and increased blood flow into the corpus cavernosum. This rapid blood inflow leads to compression of the venous network and reduction of venous outflow, thereby increasing the intracavernous pressure of the penis and leading to erection (11, 12). When cGMP is degraded by phosphodiesterase type 5 (PDE5) enzymes, the erection eventually subsides (13). Patients with mild ED can be treated with medications such as oral PDE5 inhibitors (PDE5is) (14). Although PDE5i is now widely considered a first-line treatment for ED, up to 35% of patients do not respond to it (15). Patients with moderate to severe ED need to be treated with cavernosal active drug injection, vacuum erection device, and even prosthesis implantation (14). These methods are often not acceptable to patients because they are invasive, complex, and expensive (16). Therefore, a more effective treatment for ED is required.

L-arginine is an amino acid compound obtained by the human body from diet and endogenous metabolism. L-arginine is the substrate of many enzyme pathways involved in the regulation of vascular tone, immune activation, and cell growth (17). As a substrate of NOS, it is catalyzed by NOS to convert to NO. Studies have shown that oral administration of L-arginine can improve sperm quality and erectile function in male infertility patients (18). Pycnogenol, an extract from the bark of French Marine pine, is a substance composed of proanthocyanidins, monocatechin, paclitaxel, phenolic acid, and other flavonoids. Pycnogenol is a very strong antioxidant (19), which increases NO synthesis by catalyzing E-NOS (19, 20). When Pycnogenol ® is administered in combination with L-arginine, the substrate of E-NOS, a synergistic effect of NO production can be achieved (21). Clinically, the combination of Pycnogenol ® and L-arginine (PAL) has been evaluated in several randomized controlled trials (RCTs). Existing research results showed that the combined treatment of PAL had inconsistent results in improving the sexual function index of patients with ED.

Because of the limited number of studies available on the combination of PAL in patients with ED, there is a lack of uniform evaluation criteria for efficacy and safety of combination therapy. Therefore, it is necessary to study the efficacy and safety of the combination of PAL in the treatment of patients with ED. To our knowledge, this study is the first meta-analysis to compare their therapeutic effects. It is expected to provide more reliable evidence for the efficacy and safety of the combination of PAL in patients with ED.

## Methods

### Study registration

This study was registered with Prospero (CRD42023411781) and was designed according to the Preferred Reporting Items for Systematic Reviews and Meta-Analysis (PRISMA, 2020) guidelines (22).

### Databases and search strategies

A total of seven commonly used databases were retrieved from the time the database was established until 15 February 2023, specifically PubMed, Embase, Web of Science, The Cochrane Library, Chinese National Knowledge Infrastructure (CNKI), VIP database, and Wanfang database. In addition, unpublished gray literature and references cited in eligible studies were retrieved. We use a search strategy that combines Medical Subject Headings (MeSH) terms with free-text words. For example, when conducting a search in PubMed, the following terms are used: (((“Arginine”[Mesh]) OR (Arginine[Title/Abstract])) AND ((“Pycnogenols” [Supplementary Concept]) OR (((((Pycnogenols[Title/Abstract]) OR (Pinus pinaster bark extract[Title/Abstract])) OR (French maritime pine bark extract[Title/Abstract])) OR (maritime pine bark extract[Title/Abstract])) OR (Pycnogenol[Title/Abstract])))) AND (“Erectile Dysfunction”[MeSH Terms] OR “Erectile Dysfunction”[Title/Abstract] OR “male impotence”[Title/Abstract] OR “male sexual impotence”[Title/Abstract] OR “Impotence”[Title/Abstract]).

### Selection exclusion criteria

Articles that fulfilled the following criteria were selected for this study: RCTs in humans with the parallel group or crossover design; the experimental group received a combination of PAL, whereas the control group received placebo at similar intervals; measuring mean changes of outcome indicators, such as international index of erectile function (IIEF) scores (erectile domain), orgasmic function scores, sexual desire scores, intercourse satisfaction scores, overall satisfaction scores, and testosterone (T); data were presented as mean [± standard deviation (SD)] or with 95% confidence intervals (CIs) for the placebo and intervention groups; involving human RCTs published in the English language; reviews, conference abstracts, and studies with unavailable full text were excluded.

### Study selection and data extraction

Two researchers (YT and QZ) extracted articles on the basis of inclusion and exclusion criteria, respectively. Disagreements between the two researchers were resolved by a third reviewer (QC). Excel tables are used to extract and save data. We will extract the following data from the included meta-analyses: first author, time of publication, treatment, country, the total number of cases, number of cases in the experimental group, number of cases in the control group, method of specific treatment for each group, time of therapy, adverse reaction, age, and Body Mass Index (BMI). For articles that lacked sufficient data or where data were not available, we contacted the corresponding author by e-mail for more details. If not, we analyze only the available data.

### Quality assessment of studies

The risk of bias for the included studies was independently assessed by two authors (YT and QZ), and disagreements were resolved by a third reviewer (QC). The methodological quality and bias of all eligible studies were assessed by two independent evaluators using the Cochrane Collaboration’s bias risk tool and standard Excel sheets. Any ambiguity or discrepancy in this course was resolved through discussion and participation of a third person. Using the following seven criteria, we assessed the quality of studies: (1) random sequence generation, (2) allocation concealment, (3) blinding of participants and personnel, (4) blinding of outcome assessment, (5) incomplete outcome data, (6) selective reporting, and (7) other probable sources of risk biases.

### Data synthesis

The data analysis was performed using the RevMan 5.4 software. Continuous variables were presented as mean difference (MD) and 95% CI. The standard MD is used for the same measure with different measurement methods. We assessed statistical heterogeneity between studies using Cochran Q and I^2^ statistics (23). This indicates a significant heterogeneity if the Q-statistics > the df with a p-value of < 0.05. P < 0.05 was considered statistically significant. A random-effects model was used in the study to reduce the effect of heterogeneity.

## Results

### Search results

**Figure 1:**
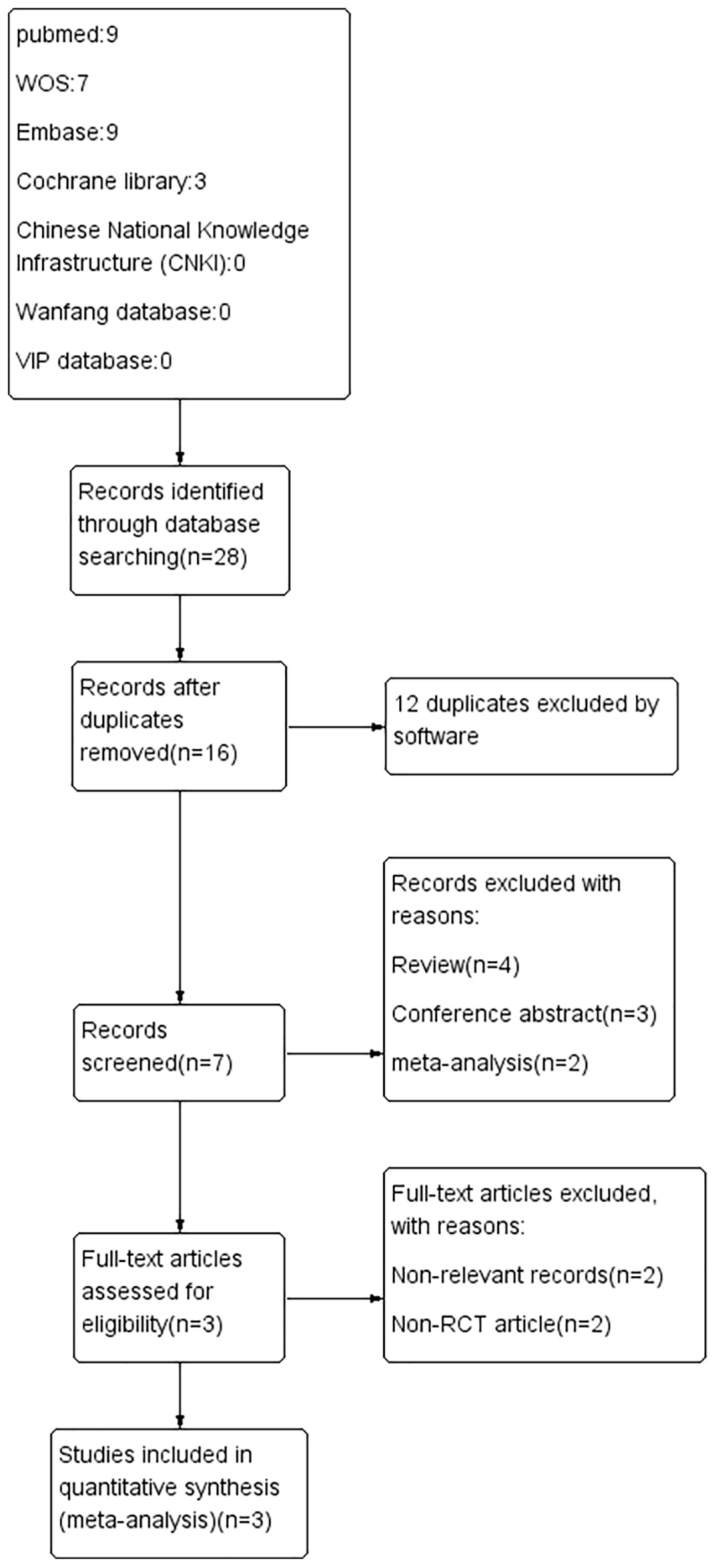
The flowchart of the study selection process.

A total of 25 articles were initially identified through an electronic database search. We import our search results into EndNote V.X9 software. After removing duplicates, reviewing titles and abstracts, and reading through the full text, finally, three articles (24–26) were included in the systematic review and meta-analysis, involving 184 patients, including 93 in the placebo group and 91 in the intervention group. Details on findings indicated that one study had low bias risk, one study had unclear bias risk, and one study had high bias risk. The study selection process is shown in the PRISMA flow chart (**Figure 1**). The characteristics included in the clinical trials and more detailed information are shown in **Table 1**.

**Table 1:** Baseline characteristics of included studies.

Authors	Country/publication year	Design	Follow-up	Total cases	Sample size		Treatment	Age (years)		Blood pressure (mmHg)		BMI (kg/m2)		ED severity	Adverse reaction
					Experimental	Control		Experimental	Control	Experimental	Control	Experimental	Control		
Andrea Ledda et al. (20)	Italy/2009	RCT, double-blindstudy	6 months	124	54	57	①	44.5 ± 4 (30–50)	44 ± 4 (30–50)	138.9 ± 8.0/none	137.0 ± 6.8/None	24.6 ± 2.6	22.3 (2.6)	Mild to moderate	None
Hiromitsu Aoki et al. (21)	Japan/2011	RCT, double-blind study	2 months	24	12	12	②	51.4 ± 9.0	50.6 ± 7.5	128.4 ± 12.9/80.8 ± 10.0	125.7 ± 13.9/76.6 ± 10.2	24.4 ± 2.0	23.4 ± 2.9	Mild to moderate	None
R Stanislavov et al. (22)	Bulgari/2008	RCT, double-blind crossover study	1 month	50	25	25	③	36.8 (30–50)	37.2 (30–50)	134.6 ± 7.1/85.8 ± 4.5	132.4 ± 7.7/86.0 ± 5.0	26.2	25.9	Mild to moderate	None

① Each PAL tablet contained 20 mg of Pycnogenol® plus 700 mg of L–arginine aspartate. In the placebo tablets, dicalcium phosphate replaced the active components. The standard is two tablets in the morning and two in the evening.

② Subjects were instructed to take a supplement (Pycnogenol® at 60 mg/day, L‐arginine at 690 mg/day, and aspartic acid at 552 mg/day) or an identical placebo for 8 weeks.

③ The daily dose of PAL corresponds to an intake of 80 mg of Pycnogenol® and 3 g of L-arginine aspartate. The standard is two tablets in the morning and two in the evening.

### Quality assessment

**Figure 2:**
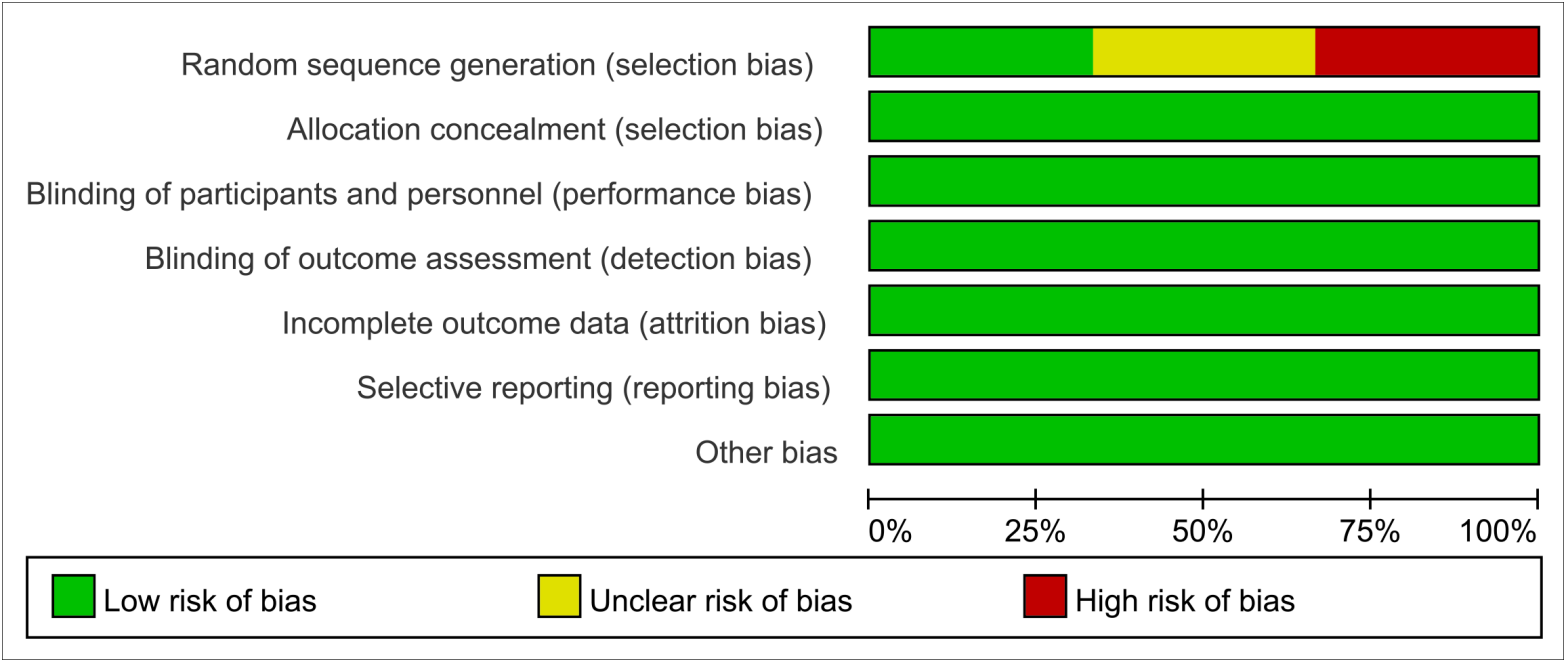
Methodological quality of the included studies (risk of bias).

**Figure 3:**
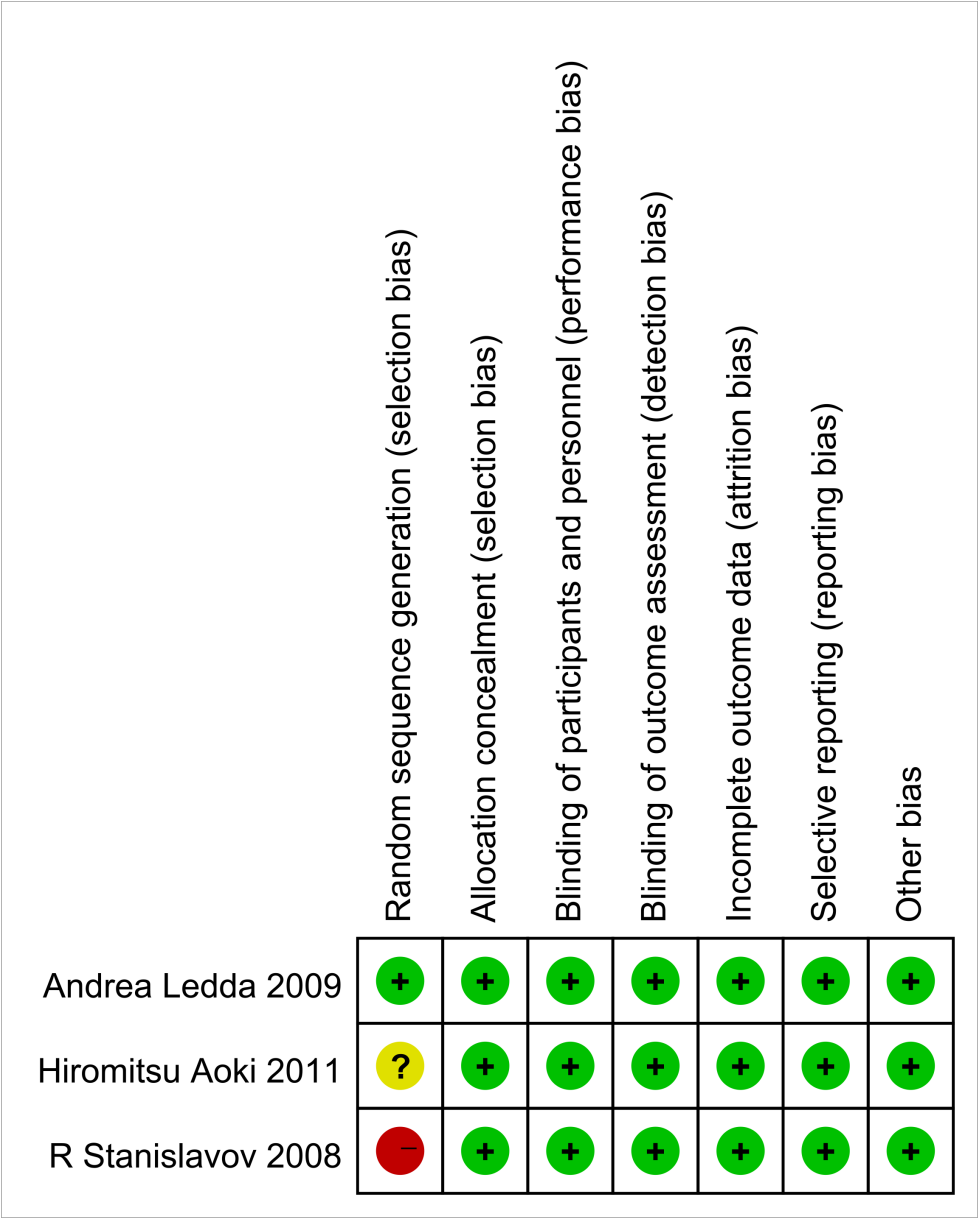
Methodological quality of the included studies (risk of bias).

The methodological quality and bias of all the eligible studies were assessed using the Cochrane Collaboration’s bias risk tool and standard Excel forms. The risk of bias in the authors’ judgments for each of the included studies is summarized in **Figures 2**, **3**.

### Main outcomes

**Figure 4:**
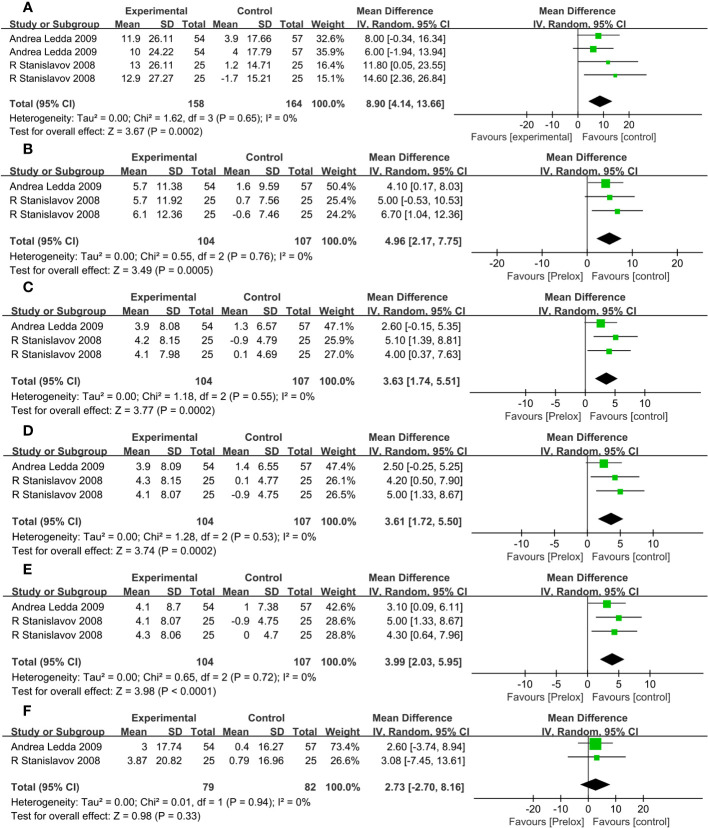
**(A)** IIEF scores (erectile domain). **(B)** Intercourse satisfaction. **(C)** Orgasmic function. **(D)** Overall satisfaction. **(E)** Sexual desire. **(F)** Testosterone (T).

Effect of combined treatment with PAL on outcome indexes are presented in **Figure 4**. A random-effects model was used to pool the results.

#### Effects on IIEF scores (erectile domain)

Among all the included clinical trials, a total of two studies (24, 26) reported IIEF scores (erectile domain) in two groups of patients. The risk of heterogeneity between studies was low (P_he_= 0.65, I^2 ^= 0%), and a random-effects model was used to combine the effect sizes of clinical studies. Meta-analysis showed that the combined treatment group was superior to the placebo group in improving IIEF scores (erectile domain) (MD, 8.9; 95% CI, 4.14 to 13.66; P = 0.0002).

#### Effects on intercourse satisfaction scores

Two studies (24, 26) reported intercourse satisfaction. The risk of heterogeneity between studies was low (P_he_= 0.76, I^2 ^= 0%), and a random-effects model was used to combine the effect sizes of clinical studies. Meta-analysis showed that, compared with the placebo group, the combination group is superior in improving the intercourse satisfaction scores (MD, 4.96; 95% CI, 2.17 to 7.75; P = 0.0005).

#### Effects on orgasmic function scores

Of all the included clinical trials, a total of two studies (24, 26) reported orgasmic function. The risk of heterogeneity between studies was low (P_he_= 0.55, I^2 ^= 0%), and a random-effects model was used to combine the effect sizes of clinical studies. Meta-analysis showed that the combined treatment group improved the orgasmic function scores more than the placebo group (MD, 3.63; 95% CI, 1.74 to 5.51; P = 0.0002).

#### Effects on overall satisfaction scores

Overall satisfaction was reported in two studies (24, 26). There was a low risk of heterogeneity among studies (P_he_= 0.53, I^2 ^= 0%). Random-effects model was used to combine the effect sizes of clinical studies. Meta-analysis showed a significant increase in overall satisfaction scores in the combined treatment group compared with that in the placebo group (MD, 3.61; 95% CI, 1.72 to 5.50; P = 0.0002).

#### Effects on sexual desire scores

Among all the included clinical trials, sexual desire was reported in two studies (24, 26). There was a low risk of heterogeneity among studies (P_he_= 0.72, I^2 ^= 0%). Random-effects model was used to combine the effect sizes of clinical studies. Meta-analysis showed that sexual desire scores in the combined treatment group increased significantly compared with that in the placebo group (MD, 3.99; 95% CI, 2.03 to 5.95; P < 0.0001).

#### Effects on testosterone

Two studies (24, 26) described the variation of T. There was a low risk of heterogeneity among studies (P_he_= 0.94, I^2 ^= 0%). Random-effects model was used to combine the effect sizes of clinical studies. Meta-analysis showed that there was no statistical significance in the change of T between the two treatment groups (MD, 2.73; 95% CI, −2.70 to 8.16; P = 0.33).

### Subgroup analysis

**Figure 5:**
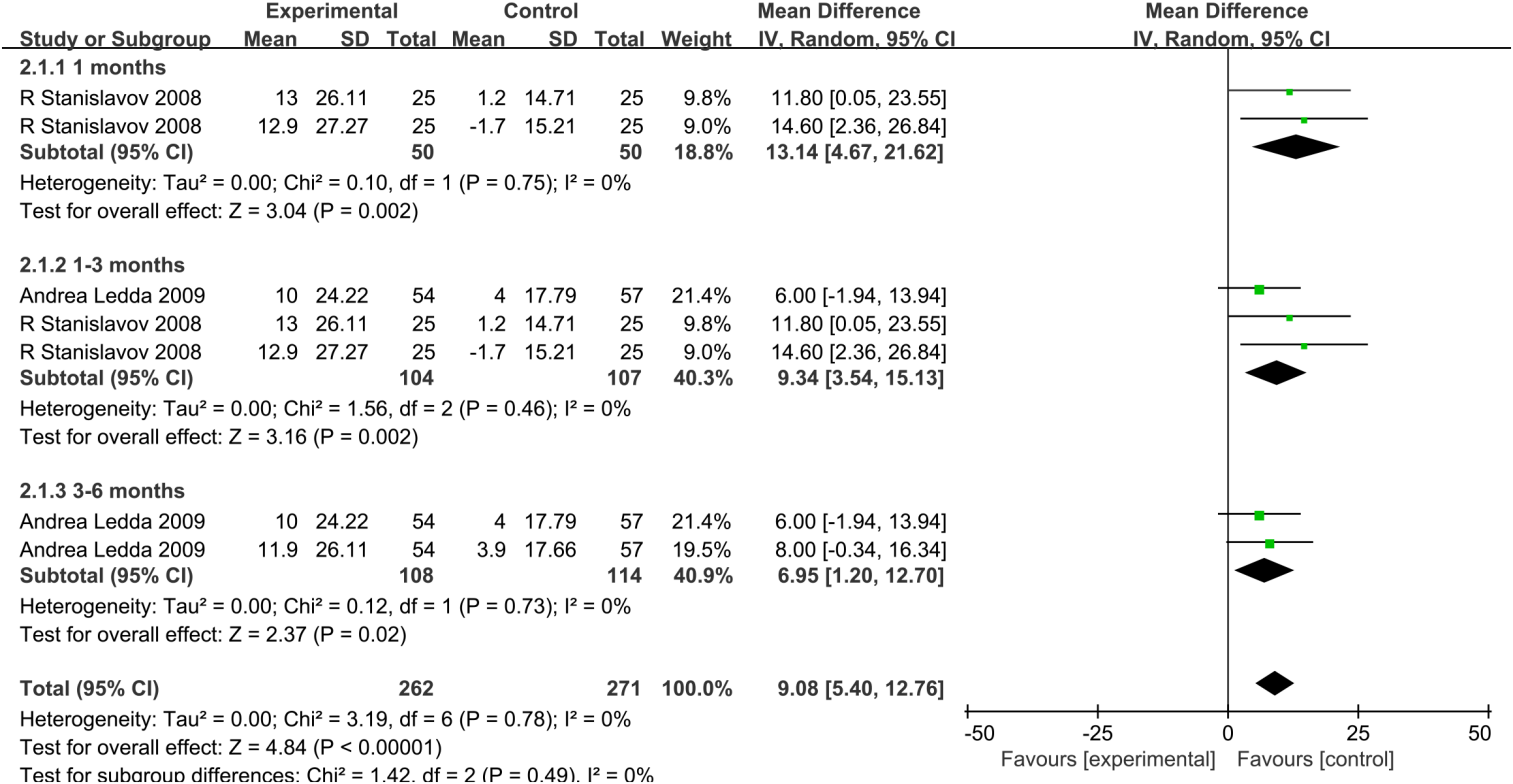
Subgroup analysis.

We performed a subgroup analysis of IIEF scores (erectile domain) on the basis of intervention duration (**Figure 5**) Our results showed a statistically significant difference between the combined treatment group and the placebo group when intervention duration was 1 month (P_he_= 0.75, I^2 ^= 0%; MD, 13.14; 95% CI, 4.67 to 21.67; P = 0.002). When the intervention duration was 1–3 months, there was a significant difference between the two groups (P_he_= 0.46, I^2 ^= 0%; MD, 9.34; 95% CI, 3.54 to 15.13; P = 0.002). When the duration of intervention was 3–6 months, the efficacy of the combination treatment group was superior to that of the placebo group (P_he_= 0.73, I^2 ^= 0%; MD, 6.95; 95% CI, 1.20 to 12.67; P = 0.02). In conclusion, there was no significant difference in the influence of intervention duration on IIEF scores (erectile domain).

### Bias risk

**Figure 6:**
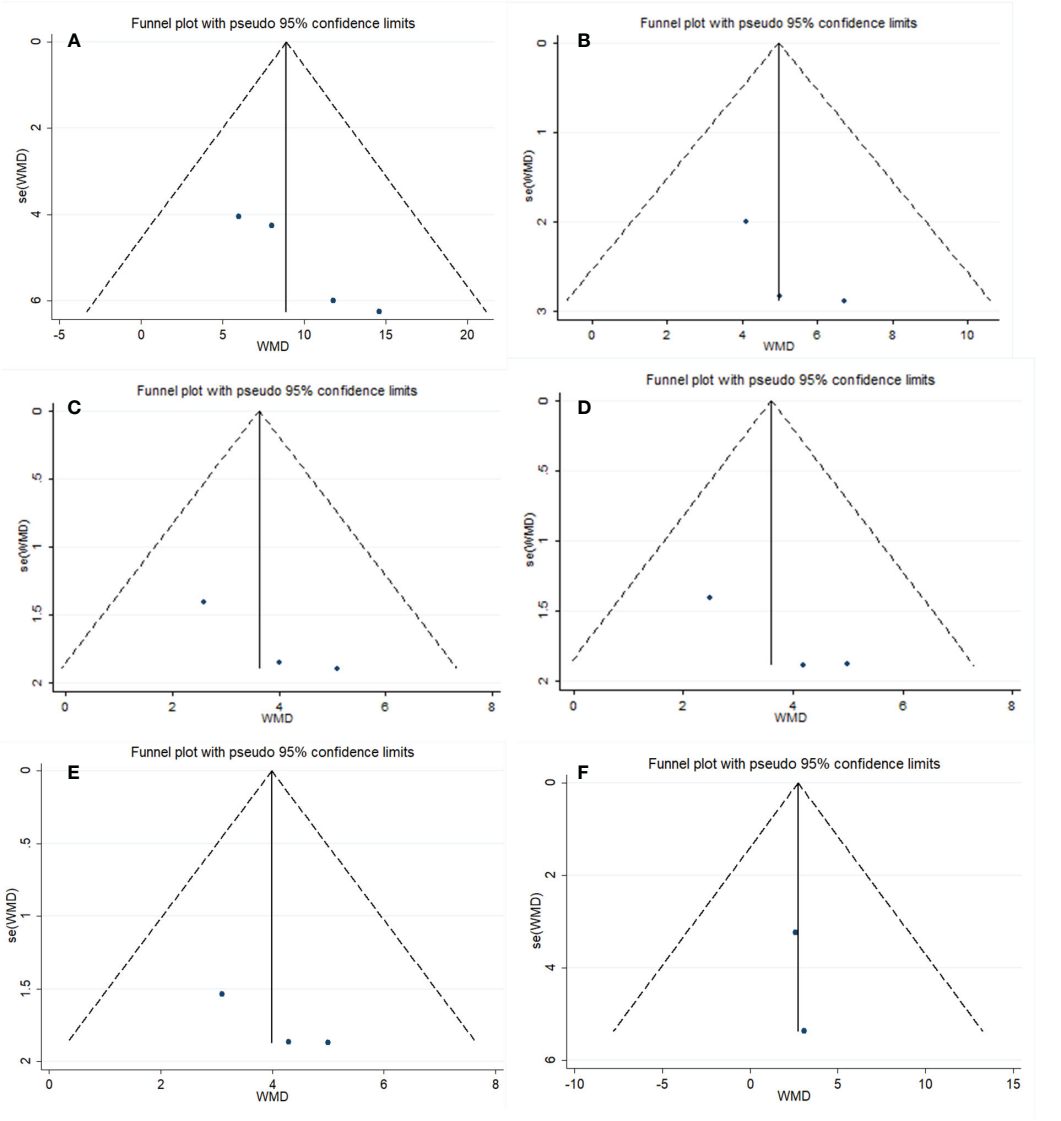
Funnel diagram. **(A)** IIEF scores (erectile domain). **(B)** Intercourse satisfaction. **(C)** Orgasmic function. **(D)** Overall satisfaction. **(E)** Sexual desire. **(F)** Testosterone (T).

**Figure 7:**
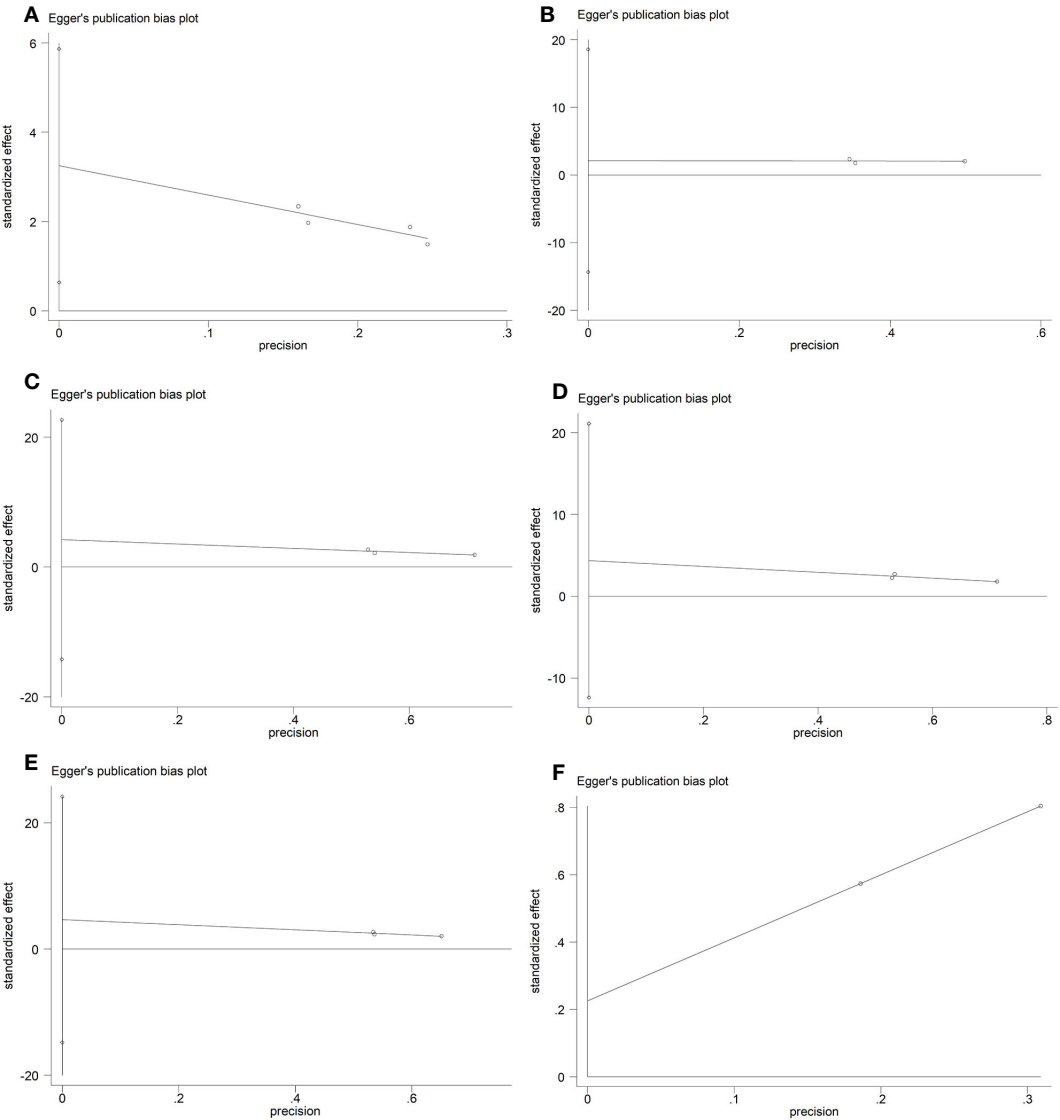
Egger’s test. **(A)** IIEF scores (erectile domain). **(B)** Intercourse satisfaction. **(C)** Orgasmic function. **(D)** Overall satisfaction. **(E)** Sexual desire. **(F)** Testosterone (T).

The funnel diagram and Egger’s test results produced by Stata 12 software are shown in **Figures 6**, **7**.

The results of the funnel plot, the Egger’s test (P = 0.033), and Begger’s test (P = 0.089) show that the analysis for Erectile domain has no publication bias. The results of the funnel plot, the Egger’s test (P = 0.351), and Begger’s test (P = 0.296) show that the analysis for intercourse satisfaction has no publication bias. The results of the funnel plot, the Egger’s test (P = 0.211), and Begger’s test (P = 0.296) show that the analysis for orgasmic function has no publication bias. The results of the funnel plot, the Egger’s test (P = 0.187), and Begger’s test (P = 1.000) show that the analysis for overall satisfaction has no publication bias. The results of the funnel plot, the Egger’s test (P = 0.202), and Begger’s test (P = 0.296) show that the analysis for sexual desire has no publication bias. The results of the funnel plot and Begger’s test (P = 1.000) show that the analysis for T has no publication bias. Perhaps because only two studies were included in the T outcome index, the P-value of Egger’s test was not displayed in the software.

## Discussion

ED is a disorder common in men and has extremely serious negative effects on the quality of life of men worldwide (27). Vascular, hormonal, neurological, psychological, lifestyle, and aging factors all play a role in the development of ED, and, when the role of one of them is unusual, all of them can lead to the occurrence of ED (12, 28). The mechanism of erectile process has been extensively studied, and major breakthroughs have been made in many complex molecular pathways (29). This study compared the efficacy and safety of daily concomitant administration of PAL or oral placebo in patients with ED. The main observation indicators of this study were IIEF scores (erectile domain), whereas the secondary observation indicators were overall satisfaction scores, intercourse satisfaction scores, sexual desire scores, orgasmic function scores and T. The results showed that the combined treatment group showed a significant advantage in improving the index of sexual function compared with the placebo group. However, there was no significant difference in raising T levels between the two groups. It has been reported that the combination of PAL may increase T concentration (24). This may be due to the difference in intervention duration between the two groups affecting the statistical efficacy. Therefore, more and better designed clinical trials are needed for further investigation. All three studies included middle-aged and elderly patients with mild to moderate ED. No adverse events were reported in any of the three studies. In summary, PAL may be safe and effective in improving sexual function in patients with mild to moderate ED.

One study (25), which could not be combined with other studies because of the use of the IIEF-5 and salivary T assessment, showed an improvement in total IIEF-5 scores compared with placebo after 8 weeks of PAL intake. There were also significant improvements in “hardness of erection” and “satisfaction with sexual intercourse.” Salivary T was slightly elevated but not statistically significant. It consistent with our results. One self-controlled before and after trial (21) reported that oral administration of PAL significantly improved sexual function in men with ED without any side effects. Another self-controlled before and after trial (18) found that, after 4 months of combined treatment with PAL, the total scores of IIEF and the international erectile function index in the orgasmic function domain were significantly improved without adverse events. However, in this study, there was no significant statistical significance in IIEF scores (erectile domain), overall satisfaction, intercourse satisfaction, sexual desire, and IIEF-5, which may be related to the low dose of oral PAL.

Several limitations should be considered in this systematic review and meta-analysis. Among the three studies included in this meta-analysis, the intervention time of each study was different and varied greatly, which may affect the statistical power. The limitations of this meta-analysis include that similar questionnaires, such as the IIEF scores and IIEF-5 scores used in the included studies, tend to be subjectively evaluated by patients, so proper objectivity of the data cannot be fully obtained. Because of searching Chinese database and screening out zero articles, it is proved that this kind of research is lacking in China, so it is very important to carry out domestic research. The biggest limitation of this paper is that there are few articles included and limited data available for analysis. Therefore, in the future, it will be necessary to conduct more large-scale and well-designed trials to confirm the observations on the efficacy and safety of the combination of PAL for ED.

## Conclusion

Together, the above findings indicated that the combined treatment of PAL had a significant effect on improving sexual function in aged patients with mild to moderate ED. We hope that this study will provide clinicians with more options for treating patients with ED.

## Data availability statement

The original contributions presented in the study are included in the article/**Supplementary Material**, further inquiries can be directed to the corresponding author.

## Author contributions

YT and QC contributed to the conception and design of the study. Data collection and extraction were carried out by YT and QZ. WL, ML, and QL planned the data evaluation and statistical analysis. The first draft of the manuscript was written by YT. QC provided guidance and resolved disagreements during this process. All authors contributed to the article and approved the submitted version.
