# Supplementary material for: Efficacy of L-arginine and Pycnogenol ® in the treatment of male erectile dysfunction: a systematic review and meta-analysis
Source: Front Endocrinol (Lausanne). 2023 Oct 4;14:1211720. doi: 10.3389/fendo.2023.1211720 (PMC10614297; doi:10.3389/fendo.2023.1211720)
Supplement: Supplementary file 1 [file DataSheet_1.pdf]

Pubmed：
((("Arginine"[Mesh]) OR (Arginine[Title/Abstract])) AND (("pycnogenols" [Supplementary Concept]) OR (((((Pycnogenols[Title/Abstract]) OR (Pinus pinaster bark extract[Title/Abstract])) OR (French maritime pine bark extract[Title/Abstract])) OR (maritime pine bark extract[Title/Abstract])) OR (Pycnogenol[Title/Abstract])))) AND ("Erectile Dysfunction"[MeSH Terms] OR "Erectile Dysfunction"[Title/Abstract] OR "male impotence"[Title/Abstract] OR "male sexual impotence"[Title/Abstract] OR "Impotence"[Title/Abstract])
Web of Science：
TS=(Erectile Dysfunction OR male impotence OR male sexual impotence OR Impotence) AND TS=(Arginine) AND TS=(pycnogenols OR Pinus pinaster bark extract OR French maritime pine bark extract OR maritime pine bark extract OR Pycnogenol)
Embase：
#1 'erectile dysfunction':ab,ti OR 'male impotence':ab,ti OR 'male sexual impotence':ab,ti OR impotence:ab,ti
#2 arginine:ab,ti
#3 pycnogenols:ab,ti OR 'pinus pinaster bark extract':ab,ti OR 'french maritime pine bark extract':ab,ti OR 'maritime pine bark extract':ab,ti OR pycnogenol:ab,ti
#1 AND #2 AND #3
Cochrane library：
#1 MeSH descriptor: [Erectile Dysfunction] explode all trees
#2 (Erectile Dysfunction OR male impotence OR male sexual impotence OR Impotence):ti,ab,kw
#3 #1 or #2
#4 MeSH descriptor: [Arginine] explode all trees
#5 (Arginine):ti,ab,kw
#6 #4 or #5
#7 (pycnogenols OR Pinus pinaster bark extract OR French maritime pine bark extract OR maritime pine bark extract OR Pycnogenol):ti,ab,kw
#8 #3 and #6 and #7
Chinese National Knowledge Infrastructure (CNKI):
（主题：勃起功能障碍（精确））OR（篇关摘：阳痿（精确）） AND （(主题：精氨酸（精确））OR（篇关摘：精氨酸（精确））） AND （(主题：碧容健（精确））OR（篇关摘：碧萝芷（精确））OR（篇关摘：松树皮提取物（精确））OR（篇关摘：松树皮萃取物（精确）））
Wanfang database:
主题:(("勃起功能障碍" or "阳痿") and ("精氨酸") and ("碧萝芷" or "碧容健" or "松树皮提取物" or "松树皮萃取物"))
VIP database:
M=(勃起功能障碍 OR 阳痿) AND M=(精氨酸) AND M=(碧萝芷 OR 碧容健 OR 松树皮提取物 OR 松树皮萃取物)
